# Short-Term Vegetation Restoration Enhances the Complexity of Soil Fungal Network and Decreased the Complexity of Bacterial Network

**DOI:** 10.3390/jof8111122

**Published:** 2022-10-25

**Authors:** Hengkang Xu, Chao Chen, Zhuo Pang, Guofang Zhang, Juying Wu, Haiming Kan

**Affiliations:** Institute of Grassland, Flowers and Ecology, Beijing Academy of Agriculture and Forestry Sciences (BAAFS), No. 9 Shuguang Garden Middle Road, Haidian District, Beijing 100097, China

**Keywords:** revegetation, soil microfungi, community diversity, illumina sequencing, molecular ecological networks

## Abstract

Different vegetation restoration methods may affect the soil’s physicochemical properties and microbial communities. However, it is not known how the microbial network’s complexity of the bacterial and fungal communities respond to short-term vegetation restoration. We conducted a short-term ecological restoration experiment to reveal the response of the soil’s microbial community and microbial network’s stability to initial vegetation restoration during the restoration of the degraded grassland ecosystem. The two restoration methods (sowing alfalfa (*Medicago sativa*, AF) and smooth brome (*Bromus inermis*, SB)) had no significant effect on the alpha diversity of the fungal community, but the SB significantly increased the alpha diversity of the soil surface bacterial community (*p* < 0.01). The results of NMDS showed that the soil’s fungal and bacterial communities were altered by a short-term vegetation restoration, and they showed that the available phosphorus (AP), available potassium (AK), and nitrate nitrogen (nitrate-N) were closely related to changes in bacterial and fungal communities. Moreover, a short-term vegetation restoration significantly increased the complexity and stability of fungi ecological networks, but the opposite was the case with the bacteria. Our findings confirm that ecological restoration by sowing may be favorable to the amelioration of soil fungi complexity and stability in the short-term. Such findings may have important implications for soil microbial processes in vegetation recovery.

## 1. Introduction

Most ecosystems have experienced massive degradation as a result of the increasing influence of human activities, climate change, and unsustainable land use around the world [1,2,3]. Ecosystem degradation often leads to declines in the biodiversity and ecosystem functioning [4,5], which is a process during which different ecosystem components interact and affect each other [6]. Thus, the degradation of a few components could potentially change the state of others and lead to holistic ecosystem degradation [7].

It is difficult to work to restore the degraded ecosystem by natural processes alone [8]. Efforts to restore biodiversity and ecosystem functioning primarily focus on the aboveground plant community [9,10,11]. In northern China, *Medicago sativa* L. (alfalfa) and *Bromus inermis* Leyss. (smooth brome) are widely used as a community building species for ecological restoration [10,12,13]. Alfalfa, a high-quality perennial legume, could improve the soil’s texture and nutrients in a low degree degradation [14,15]. In addition, unlike the fibrous roots of smooth brome, alfalfa has a deep rooting system, and the physicochemical properties in deep soil could be affected by alfalfa planting [16,17]. For example, it was found that the alfalfa-based systems had more total nitrogen and organic carbon in the deep soil than did the grain-based systems [18]. As degraded ecosystems are seeded with new plants, litter, residual roots, and root exudates from existing plants can alter the surrounding environment and facilitate the construction of the microbial communities within soil [19]. However, the microbial diversity and community structure at different soil depths are affected by the short-term sowing of plants with different root characteristics because such plants are not fully understood.

Soil microbial communities (bacteria and fungi) regulate global biogeochemical cycles and respond rapidly to changes in the soil’s microenvironment [20,21]. Microorganisms have an extremely high reproduction rate and richness, which allows the microbial community to have the ability to respond to environmental changes in time [20,21,22,23]. Additionally, the diversity and composition of bacterial and fungal communities plays an irreplaceable role in decomposition and nutrient cycling [24,25]. Previous studies showed that significant differences existed in the relative abundance of bacteria and fungi after restoration [10,26,27]. For example, sown treatments decreased the diversity of fungi and bacteria through decreasing the plant diversity and increasing the soil’s moisture [10]. In contrast, the short-term planting of some species resulted in an increase in the total microbial community richness and diversity [28]. Hence, it remains unclear how the diversity of bacteria and fungi changes after a short-term revegetation. Soils host the microorganisms, and the differences in its physicochemical properties, in turn, change the microbial diversity and community [29]. For example, the soil’s pH, soil water content (SWC), soil organic carbon (SOC), and total nitrogen (TN) have been widely reported to be key factors affecting the bacterial and fungal communities [30,31,32]. In addition, bacterial and fungal community composition was significantly influenced by the available phosphorus (AP) and the available potassium (AK) in subtropical soil [33]. Therefore, the degradation of the microbial communities is closely related to changes in the soil’s physicochemical and biological conditions during the ecological restoration of degraded areas [34,35]. However, it is not clear which of the soil’s physical and chemical properties drive the change in the microbial community’s structure caused by the short-term reseeding for vegetation restoration. 

Recently, microbial network analysis has been widely used by microbial ecologists and the results reveal the interrelationships, or co-occurrence patterns, between microorganisms in various environments [36,37,38]. Complicated interconnections between microorganisms can be represented as co-occurrence networks with microbial taxa as nodes and their relationships as links [39,40]. Moreover, microbial diversity and interactions between taxa can vary through time [41], space [38], or environments [42]. This indicates that the interaction between microbial species can be combined with species diversity to better understand the response of microbial communities to the environment. The next frontier is to go beyond just assessing the role of univariate microbial diversity and investigate how changes in the complexity of interconnectivity among co-occurring microbes impacts the variation in ecological processes [38]. However, to the best of our knowledge, the effect of the short-term ecological restoration of different plant types on the microbial network’s complexity is unclear.

Here, we established field experiment investigating the influence of two restoration plants with different root characteristics (*Medicago sativa* L and *Bromus inermis* Leyss) on the soil microbial community’s diversity and microbial network’s complexity in a degraded wasteland on the North China Plain. We aimed to (i) compare the structural composition and diversity of soil microbial communities in different restoration regimes and soil layers, (ii) explore the key soil physicochemical drivers of community change, and (iii) evaluate the microbial network’s complexity in different restoration regimes.

## 2. Materials and Methods

### 2.1. Site Description

The experiment started in September 2015, at the Long-term Ecological research Station of Degraded land in Yanqing District, Beijing (115°50′23″ E, 40°27′53″ N, 492 m above sea level). The mean annual precipitation is about 600 mm, mostly in June to September. The soil’s texture is intermediate between sandy soil and loam. The dominant species were *Pennisetum centrasiaticum* Tzvel. (55%), *Artemisia capillaris* Thunb (18%), and *Melica scabrosa* Trin. (15%).

### 2.2. Experimental Design

Our field experiment with a randomized design was conducted in 2015. Nine plots were established in our study, with an area of 100 m^2^ per plot, including *Medicago sativa* (AF, three plots), *Bromus inermis* Leyss (SB, three plots), and natural recovery (CK, three plots). Seeds were sown without plowing in May 2015, with the density of 200 seeds per m^2^ for each species. After the vegetation was established, the area was fenced.

### 2.3. Soil Sampling and Soil Biochemical Analyses

Three 1 m × 1 m quadrats were randomly set in each plot in 2015. Three soil cores (diameter 5 cm) were taken in each quadrat and then mixed together into one sample. Soil samples from 0–10 cm, 10–20 cm, and 20–30 cm layers were collected (total 27). The composite samples were passed through a 2 mm sieve, and any visible living plant material was removed from the sieved soil. The subsamples of the sieved soils were stored at −80 °C and 4 °C for molecular and biochemical analyses, respectively. The soil pH was measured by a potentiometer after shaking a soil water suspension (1:2.5 water/soil) for 30 min. The soil’s available phosphorus (AP) was determined using the Olsen method which involved adding 50 mL of Olsen’s reagent to 2.5 g of air-dried soil (soil–solution ratio of 1:20) and subsequently shaking it for 30 min, then the filtrate was used to determine it colorimetrically [20]. The total phosphorus (TP) was measured by the sodium hydroxide melting-molybdenum barium colorimetric method [43]. The total organic carbon (TOC) was measured with a TOC analyzer (Rapid CS Cube, Elementar, Langenselbold, Germany). The content of organic matter (OM) in the soil was calculated by multiplying the percentage of TOC by the common proportion of organic carbon in the soil (i.e., the conversion factor 1.724). The total nitrogen (TN) was measured with a C/N analyzer (Rapid CS Cube, Elementar, Langenselbold, Germany). Inorganic nitrogen (ammonium-N and nitrate-N) was extracted with 0.5 mol L^−1^ of K_2_SO_4_ and measured with a continuous flow injection analyzer (AA3 HR, SEAL Analytical GmbH, Norderstedt, Germany). The total (TK) and available potassium (AK) were measured by ammonium acetate extraction-atomic absorption spectrophotometry [44].

### 2.4. DNA Extraction, PCR Amplification, and Illumina MiSeq Sequencing

The microbial community’s genomic DNA was extracted from 0.25 g of moist soil using the PowerSoil DNA Isolation Kit (MO BIO laboratories, Carlsbad, CA, USA) following a standardized protocol. The soil DNA of each sample was extracted in triplicate and then pooled to decrease the extraction bias. The DNA extract was checked on 1% agarose gel, and the DNA concentration and purity were determined with a NanoDrop 2000 UV-Vis spectrophotometer (Thermo Scientific, Wilmington, NC, USA). The bacterial 16S rRNA gene was amplified with the primers 338F_806R and the fugal ITS region was amplified with the primers ITS1F_ITS2 by an ABI GeneAmp^®^ 9700 PCR thermocycler (ABI, Los Angeles, CA, USA). To profile the soil bacterial communities, we amplified the V3–V4 hypervariable region of the 16S rRNA gene with the primer sets 338F (5′-ACTCCTACGGGAGGCAGCAG-3′) and 806R (5′-GGACTACHVGGGTWTCTAAT-3′) [20,45]. For the fungal communities, we amplified the ITS region with the primers sets ITS1-F (5′-CTTGGTCATTTAGAGGAAGTAA-3′) and ITS2 (5′-TGCGTTCTTCATCGATGC-3′) [20,46]. The PCR reactions for both communities are detailed in the Supplementary Methods. The purified amplicons were pooled in equimolar and paired-end sequences (2 × 300) on an Illumina MiSeq platform according to standard protocols of Majorbio Bio-Pharm Technology Co., Ltd. (Shanghai, China).

After demultiplexing and quality-filtering, the acquired raw 16S rRNA and internal transcribed spacer (ITS) sequence data were sorted into valid reads using the Quantitative Insights into Microbial Ecology (QIIME; version 1.9.1; http://qiime.org/index.html, accessed on 19 September 2022) pipeline with the criteria detailed in the Supplementary Methods. The ribosomal database project (RDP) classifier was used to assign 16S rRNA and ITS gene sequences to taxonomic groups based on the SILVA database (version 132) and the UNITE fungal ITS database (version 7.2), respectively, at an identity threshold of 75%.

### 2.5. Microbial Co-Occurrence Network Construction

To reduce rare OTUs and those with a low abundance from the dataset, data filtering was conducted prior to the network construction. All OTUs were removed that comprised <0.01% of the total number of reads. At the same time, we selected the top 200 taxa with an OTU abundance for analysis. All the pairwise correlation scores of the co-occurrence network were obtained by calculating both the Spearman correlation and Jaccard dissimilarity measures, using an approach based on the random matrix theory (RMT) [42]. The network patterns were plotted with Gephi and were visualized by the Frucherman Reingold algorithms. To ensure that the derived network is non-random and scale-free, these networks were evaluated against their networks (100 randomized versions) with the same number of vertices and edges [38]. The obtained site-level network meta-matrices were then used to sub-set network matrices for each sampling plot by preserving the OTUs present within the plot and all the edges among them in the site-level network [47]. The following topological parameters (which indicate linkage density), the node and link numbers, average neighbors, connectance (i.e., the proportion of realized links from all possible connections in the network), and linkage density (links per OTU), were tightly correlated, thus linkage density was used to denote the network complexity index [38,47,48].

### 2.6. Statistical Analysis

We used microbial OTU richness as the metrics of, and calculated, the microbial α-diversity. A one-way ANOVA was used to determine the significance of the effects of different restoration regimes on the following response of the soil variables and the diversity of the bacterial and fungal communities. The Kruskal–Wallis test was used to determine the significance of phyla and classes of bacterial and fungal communities. Before conducting the ANOVA, the normality and homoscedasticity of the residues were verified by the Kolmogorov–Smirnov test and Levene’s test, respectively. Significant differences were determined at the 0.05 and 0.01 levels. All data are presented as mean values ± standard error (SE). The effects of different restoration regimes on the bacterial and fungal communities’ structures were further tested by non-metric multidimensional scaling (NMDS) using an OTUs-based Bray–Curtis. Spearman correlation analysis was used to assess the relationships between the relative abundance of bacterial and fungal taxa and soil properties (i.e., the soil physicochemical properties and soil moisture). Both the NMDS and Spearman analyses were performed using the VEGAN package [38] in R 3.5.2 (R Development CoreTeam, Vienna, Austria, 2015). We calculated the abundance-based Bray–Curtis, as the metrics of beta diversity, to quantify the community compositional difference between the replicate plots of the same treatment. We assessed the relative importance of the soil’s physicochemical conditions for the beta diversity of soil microbial communities, using the linear regression method. Other statistical analyses were performed using SPSS 20.0 (IBM Corporation, Armonk, NY, USA).

## 3. Results

### 3.1. Soil Physicochemical Properties

Over the 2-year experimental period, the soil physicochemical properties changed significantly after sowing (Table 1). The soil’s OM, TN, TP, AP, AK, AN, and ammonium-N at the depth of 0–10 cm soil increased significantly (*p* < 0.05) in the alfalfa sowing treatments (AF). The soil’s pH and TK showed no significant differences between the different sowing treatments. The SB sowing treatments significantly increased the AK (Table 1; *p* < 0.05).

### 3.2. Bacterial and Fungal Community Structure and Species Diversity

**Figure 1:**
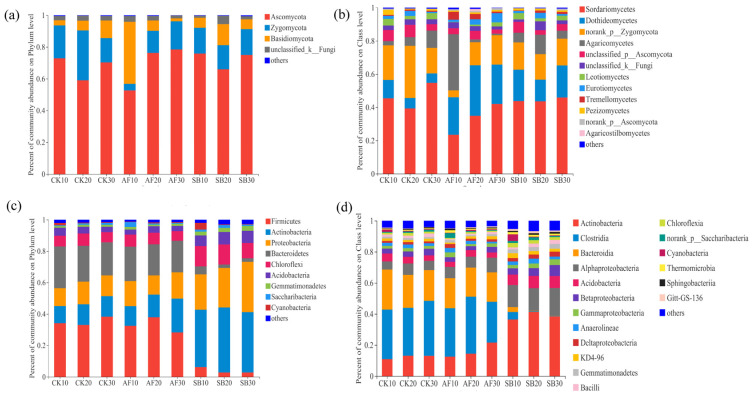
Taxonomic profiles of fungal community composition at the phylum level (**a**) and the class level (**b**) and bacterial community composition at the phylum level (**c**) and the class level (**d**) in different soil layers (0–10 cm, 10–20 cm, and 20–30 cm) under different restoration methods. Shown are group accounting for >1% of the relative abundance, while groups accounting for <1% are integrated into ‘others’. CK, natural recovery; AF, *Medicago sativa* L sowing; and SB, *Bromus inermis* Leyss sowing.

Our results show that the relative abundances of fungi and bacteria at the phylum and class level were different among the two restoration methods (Figure 1). In the fungal community analysis across all the soil samples, a total of 1,089,774 high-quality sequences were identified. Each library had 40,362 reads and a total of 2410 OTUs were obtained. Sequences that could not be classified into any known group were assigned as unclassified and groups with an average relative abundance of less than 1% were classified as ‘others’ (Figure 1). For the phylum of fungi, the relative abundance of Rozellomycota at the depth of 0–10 cm in the AF treatment was significantly larger than in the CK treatment (Figure S1a). For the class of fungi, the relative abundance of Tremellomycetes, Microbotryomycetes, and unclassified_ Rozellomycota at the depth of 0–10 cm in the AF treatment was significantly larger than in the CK treatment (Figure S1b). 

In the bacterial community analysis, across all the soil samples, a total of 706,590 high-quality sequences were identified. Each library had 26,170 reads and 5291 operational taxonomic units (OTUs) were obtained. For the phylum of bacteria, the relative abundance of Actinobacteria in the SB treatment was significantly larger than in the CK treatment, while that of Firmicutes was smaller in the SB treatment (Figure 1c and Figure S2a). For the class of bacteria, the relative abundance of Actinobacteria and Alphaproteobacteria in the SB treatment was significantly larger than in the CK treatment, while that of *Clostridia* was smaller in the SB treatment (Figure 1d and Figure S2b). 

**Figure 2:**
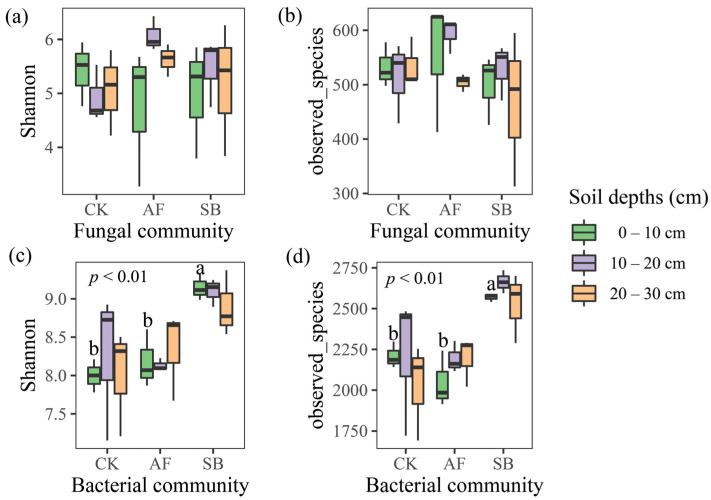
Diversity of fungi, and bacteria in different restoration methods. Shown are (**a**) Shannon–Wiener Index of fungi; (**b**) Observed_species of fungi; (**c**) Shannon–Wiener index of bacteria; and (**d**) Observed_species of bacteria. Different lowercase letters above the standard error bars indicate significant differences among the treatments (*p* < 0.01). CK, natural recovery; AF, Medicago sativa L sowing; and SB, Bromus inermis Leyss sowing.

**Figure 3:**
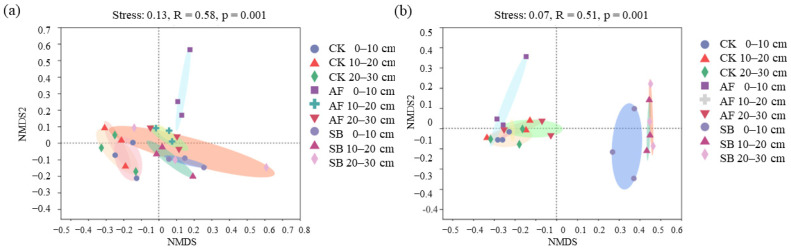
Non-metric multidimensional scaling (NMDS) of fungal community composition (**a**) and bacterial community composition (**b**) at the OTU level in in different restoration methods. CK, natural recovery; AF, *Medicago sativa* L sowing; and SB, *Bromus inermis Leyss* sowing.

There was no significant effect on the alpha diversity of the fungal community in the sowing treatment (Figure 2a,b; *p* < 0.01), but the SB treatment significantly increased the bacterial alpha diversity in the soil’s surface layer (Figure 2c,d; *p* < 0.01). The fungal and bacteria composition was further analyzed with NMDS at the OTU level. The results of NMDS showed that the soil’s fungal communities at the depth of 0–10 cm in the AF treatment were different from the other treatments (Figure 3a; stress = 0.13, *p* = 0.001) and the soil’s bacterial communities in the SB treatment was significantly altered (Figure 3b; stress = 0.07, *p* = 0.001).

### 3.3. Relationship between Environmental Variables and Community Structure of Bacteria and Fungi

**Figure 4:**
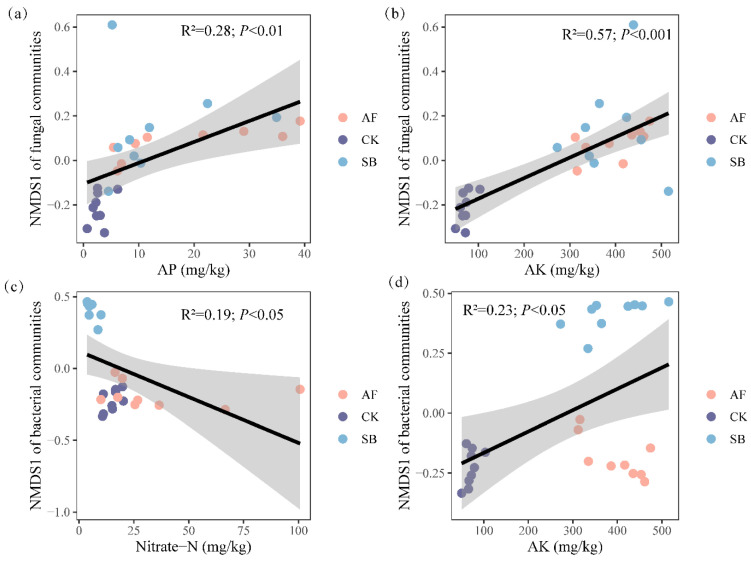
Relationship between environmental variables and community structure of bacteria and fungi. Regression analysis of AP (**a**) and AK (**b**) and NMDS of fungal communities. Regression analysis of Nitrate-N (**c**) and AK (**d**) and NMDS of bacterrial communities. AP: available phosphorus; AK: available potassium; Nitrate-N: nitrate nitrogen; grey area represents the 95% confidence interval of the linear regression.

Regression analyses showed that the AP and AK had significant positive relationships with the soil’s fungi community (Figure 4a,b; *p* < 0.01). The nitrate-N had significant negative relationships with the soil’s bacterial community, while the AK had a positive relationship with the bacterial community (Figure 4c,d; *p* < 0.05).

### 3.4. Soil Microbial Network Complexity

**Figure 5:**
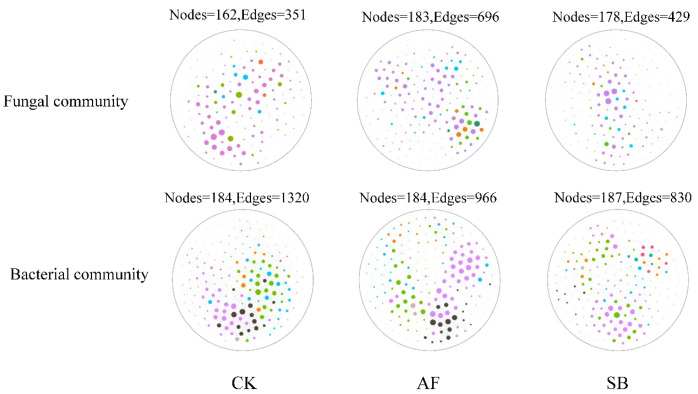
Network visualization of bacterial and fungal co-occurrence patterns in different restoration methods. Nodes indicate individual operational taxonomic units (OTUs), while edges represent a significant correlation between OTUs.

The network of the soil’s bacterial and fungal communities at each treatment demonstrated distinct co-occurrence patterns (Figure 5; Table 2). Here, we used the network topological parameters of the node and edge numbers, average neighborhood, and linkage density, to assess the complexity of the soil’s microbial network, with higher node and edge numbers and linkage density representing a greater network complexity. The complexity denoting the network properties, i.e., the numbers of nodes and edges, average neighborhood, clustering coefficient, as well as the linkage density among taxa, were the highest in the AF treatment in the fungal communities (Table 2). For the bacteria, the edge numbers and linkage density were lower in the SB treatment (Figure 5; Table 2).

## 4. Discussion

### 4.1. Impact of Vegetation Restoration on Soil Properties

Although vegetation restoration is known to improve the soil’s quality and increase the soil’s microbial activity, its effects on the soil’s nutrients remain largely uncertain because of the different restoration approach and age. Many studies, including those based on field observations [49,50,51] and meta-analyses [52], have shown that ecological restoration tends to improve the functioning of an ecosystem. In our study, we found that the soil’s properties improved after a short-term vegetation restoration, and the recovery effects depended on the restoration approach. For example, our findings show that the pH values did not change under the two different vegetation restoration modes (Table 2). This is consistent with previous findings in the southeast fringe of the Tengger Desert, China [49]. However, more studies have found that vegetation revegetation reduces the soil’s pH value in the subtropical karst region in China and degraded alkaline grassland in northeast China [53,54,55]. This may be due to a change in the soil’s pH, which depends on the soil’s texture and land use [56]. In addition, previous studies demonstrated the significant impacts of vegetation restoration improving the soil quality. For example, the organic matter, TN, and available nutrient were significantly improved after 15 years of vegetation restoration in a degraded sandy grassland in the farming–pastoral ecotone [57]. Revegetation on the desertified area increased the soil’s organic matter, available nutrients (N, P, and K), and readily oxidizable carbon (ROC) in the northern Shaanxi province of China [58]. Our findings show that OM, TN, TP, AP, AK, AN, NO_3_^−^, and NH_4_^+^ at the top of the soil (0–10 cm) were significantly increased in the AF treatment, while only NO_3_^-^ and AK were significantly increased in the SB treatment. This may be due to the fact that legumes can fix nitrogen by means of symbionts, making the fixed nitrogen available for other plants [59,60], and the soil nitrogen could be considered as a determinant of the concentration of the soil’s nutrient content and, consequently, regulate the C and P cycles [61]. Thus, compared with SB, the AF could effectively improve the fertility of the soil’s surface in a shorter time. 

### 4.2. Effects of Vegetation Restoration on the Soil Fungal Community Structures

Previous studies have found positive [62,63] or negative [47,64] effects of vegetation restoration on fungal diversity. For example, the decrease in plant diversity in the sown treatments was followed by a decline in the diversity of plant litter and root exudates; a reduction in the heterogeneity of resources may induce a reduction in the fungal diversity [10,64]. On the contrary, some studies have shown that vegetation restoration can improve fungal diversity by increasing the enzyme activities and soil nutrients [65,66,67]. Our results showed that the AF treatment significantly changed the fungal community structure at the depth of 0–10 cm but did not affect the fungal community’s species alpha diversity (Figure 2 and Figure 3). This indicated differences in their functioning, despite the equal levels of fungal diversity. In addition, the AF treatment significantly increased the relative abundance of Rozellomycota (Figure S1), and we also found that OM, TN, TP, AP, AK, AN, NO_3_^−^, and NH_4_^+^ at the top of the soil (0–10 cm) were significantly positively correlated with Rozellomycota (Figure S3). Therefore, the difference in the soil fungal community’s structure is closely related to the change in the soil’s nutrients. Our findings reinforce this idea. Regression analyses showed that AP and AK had significant positive relationships with the soil’s fungal community (Figure 4). This implies that both the AK and AP regulate the fungal community’s structure during a short-term vegetation restoration.

### 4.3. Effects of Vegetation Restoration on the Soil Bacterial Community Structures

In semi-arid climates, soils are often found in pre-degenerate states with a constrained vegetation, soil nutrient and ecosystem functionality. These limitations negatively impact soil microbial communities, which are important drivers of biogeochemical processes and strongly influence the soil’s quality [68]. Bacteria, linking soil, and plants play an important role in regulating the succession and restoration of vegetation [69]. The findings in our study showed that the SB treatment significantly increased the bacterial alpha diversity in the soil’s surface layer, while this did not occur in the AF treatment (Figure 2). The effects of vegetation restoration on the microbial diversity are controversial. A growing body of evidence suggests that the bacterial diversity in a sowing or planting area tends to be higher than in an area that recovers naturally from a disturbance [69,70,71]. On the contrary, the bacteria decreased significantly [10] and maintained a good stability [71] after the vegetation restoration. For example, a study in a mining area on the Loess Plateau in China show that, although the region has experienced about 20 years of vegetation restoration, the microbial community still maintains a good stability and lagging response to the soil’s biochemical properties [71]. In addition, revegetation by the sowing of a single species led to a reduction in the diversity of bacteria which was determined by a reduction in the plant diversity [10]. Thus, the different effects of vegetation restoration on the bacterial community’s diversity may be determined by the soil nutrient status [71,72], restoration vegetation selection [62,69,72], plant diversity level [10,71,73], and restoration time [62,73]. Previous results found that the bacterial community’s structure is closely related to the soil’s nutrients in artificial vegetation restoration [69,70]. The results of NMDS showed that the soil’s bacterial communities in the SB treatment were significantly altered (Figure 3). Importantly, however, we found that nitrate nitrogen and the AK were the main factors driving the change in the bacterial community (Figure 4c,d). This is probably because the AK was significantly negatively correlated with *Bacteroidetes* and significantly positively correlated with *Actinobacteria* and *Proteobacteria* (Figure S3). This implies that the AK regulates the bacterial community’s structure during a short-term vegetation restoration.

### 4.4. Effects of Vegetation Restoration on the Soil Microbial Network Complexity

Our results disclosed that the network properties varied throughout the vegetation restoration process. Both the AF and SB treatment increase the complexity of the fungal communities, especially the AF, while the edge numbers and linkage density of the bacterial community was reduced under the two different vegetation restoration methods (Figure 5 and Table 2). A study of vegetation restoration in southwest China in the karst region has shown that the proportion of fungal nodes in the co-occurrence network increased, while the proportion of bacterial nodes showed an opposite trend with the extension of a succession time series [74], which is consistent with our observations. This may indicate that bacteria dominated a pioneer taxon before the restoration and, as the soil’s nutrients increased, the competition of fungi, which had acquired enough organic matter, also increased [74].

The AF treatment continuously increases the nutrient release, which could increases the activity of the soil fungi, making the fungal community’s structure more complex and enhancing its resilience to environmental changes [75]. In general, the more complex and diverse the microbial community’s structure in the soil, the more stable the soil ecosystem, the higher the ecological function of the ecosystem, and thus the more obvious the buffering effect is on external environmental changes.

## 5. Conclusions

This study was conducted on a degraded wasteland on the North China Plain and examined the short-term effects of the response of the soil’s microbial community and microbial network’s stability. Different vegetation restoration methods may have different effects on the diversity and structure of the bacterial and fungal communities. Short-term vegetation can increase the complexity of the fungal networks and reduce the complexity of the bacterial networks. Such findings may have important implications for soil microbial processes in the restoration of vegetation on degraded land. The AF increased the complexity of the fungal community and maintained a relatively high complexity of the bacterial community, indicating that alfalfa as a vegetation restoration species can improve the stability and complexity of fungi and maintain the balance between bacteria and fungi, to a certain extent, in the short-term. The diversity and structure of bacteria and fungi may also be affected by their microbial enemies, such as viruses and grazing microfauna, and other soil organisms (nematodes, worms, etc.). Future experiments should focus on the underlying mechanisms and processes affecting the complexity and stability of the bacterial and fungal communities in the vegetation restoration process of degraded land.

## Figures and Tables

**Table 1:** Measured soil properties in different restoration methods.

Treatment	Soil Depth	pH	Organic Matter (mg/kg)	Total Nitrogen (mg/kg)	Total Phosphorus (mg/kg)	Total Potassium (mg/kg)	Available Phosphorus (mg/kg)	Available Potassium (mg/kg)	Available Nitrogen (mg/kg)	Ammonium Nitrogen (mg/kg)	Nitrate Nitrogen (mg/kg)
	0–10 cm	7.81 ± 0.01	6.55 ± 1.75b	472.67 ± 75.97b	486 ± 18.03cd	239 ± 7.22	2.7 ± 0.16c	72.52 ± 3.54c	36.05 ± 6.00b	16.82 ± 1.69b	5.08 ± 0.16c
CK	10–20 cm	7.81 ± 0.01	7.34 ± 1.54b	510.67 ± 89.55b	438 ± 18.19d	246 ± 9.24	1.54 ± 0.47c	61.6 ± 6.84c	37.1 ± 7.31b	15.72 ± 2.67b	5.5 ± 0.35bc
	20–30 cm	7.81 ± 0.01	6.84 ± 1.93b	449.33 ± 100.86b	443.33 ± 8.41d	230 ± 18.55	4.11 ± 1.11c	80.32 ± 11.7c	31.62 ± 7.72b	12.92 ± 1.84b	5.12 ± 0.21c
	0–10 cm	7.82 ± 0.02	13.82 ± 0.45a	1027.33 ± 20.27a	686.67 ± 8.09a	233 ± 2.08	34.75 ± 3.01a	463.12 ± 6.12a	81.78 ± 3.44a	67.95 ± 18.56a	7.03 ± 0.38ab
AF	10–20 cm	7.81 ± 0.01	8.13 ± 2.01b	654.67 ± 108.21b	551.33 ± 21.80bc	214 ± 16.13	7.22 ± 1.15bc	379.12 ± 23.77b	52.38 ± 9.23b	18.12 ± 4.86b	6.47 ± 0.34ab
	20–30 cm	7.81 ± 0.01	7.41 ± 2.04b	555 ± 125.4b	520 ± 55.51cd	248 ± 8.72	13.1 ± 4.54bc	354.45 ± 40.52b	44.8 ± 9.15b	20.55 ± 2.66b	5.75 ± 0.45bc
	0–10 cm	7.83 ± 0.02	6.83 ± 1.61b	559 ± 124.71b	507 ± 52.17cd	256 ± 25.75	13.53 ± 4.75bc	323.62 ± 27.06b	37.8 ± 7.91b	7.73 ± 1.62b	5.98 ± 0.78ab
SB	10–20 cm	7.81 ± 0.01	8.23 ± 2.03b	619.33 ± 110.27b	621 ± 25.81ab	258 ± 15.54	18.14 ± 8.41b	373.12 ± 25.6b	42.12 ± 4.81b	5.05 ± 0.43b	7.35 ± 0.65a
	20–30 cm	7.83 ± 0.01	6.51 ± 0.88b	526.33 ± 79.67b	520.33 ± 14.31cd	239 ± 3.71	6.01 ± 1.18bc	470.12 ± 23.19a	40.48 ± 7.48b	4.22 ± 0.44b	5.62 ± 0.61bc

Note: different letters (a, b, and c) within the same column indicate significant differences among restoration methods. CK, natural recovery; AF, *Medicago sativa* L sowing; and SB, *Bromus inermis* Leyss sowing.

**Table 2:** Characteristic parameters of the molecular ecological network of soil microorganisms.

Treatment	Fungal Community			Bacterial Community		
	CK	AF	SB	CK	AF	SB
Number of nodes	162	183	178	184	184	187
Number of edges	351	696	429	1320	966	830
Average neighborhood	4.33	7.61	4.82	14.35	10.5	8.87
Linkage distance	14	15	15	13	10	11
Clustering coefficient	0.39	0.47	0.43	0.54	0.53	0.51
Linkage density	2.16	3.8	2.4	7.17	5.25	4.44

Note: CK, natural recovery; AF, *Medicago sativa* L sowing; and SB, *Bromus inermis* Leyss sowing.

## Data Availability

Not applicable.

## Supplementary Materials

The following supporting information can be downloaded at: https://www.mdpi.com/article/10.3390/jof8111122/s1, Figure S1: Kruskal-Wallis test bar plot for fungal phyla (a) and classes (b) in in different restoration methods; Figure S2: Kruskal-Wallis test bar plot for bacterial phyla (a) and classes (b) in in different restoration methods; Figure S3: Spearman correlation heatmaps of environment factors, biochemical properties and bacterial gene read numbers at the phylum (a) and fungal gene read numbers at the phylum (b) level.

## References

[1] Bardgett R.D., Bullock J.M., Lavorel S., Manning P., Schaffner U., Ostle N., Chomel M., Durigan G., Fry E.L., Johnson D. (2021). Combatting global grassland degradation. Nat. Rev. Earth Environ..

[2] Liu Y.Y., Zhang Z.Y., Tong L.J., Khalifa M., Wang Q., Gang C.C., Wang Z.Q., Li J.L., Sun Z.G. (2019). Assessing the effects of climate variation and human activities on grassland degradation and restoration across the globe. Ecol. Indic..

[3] Steffen W., Richardson K., Rockstrom J., Cornell S.E., Fetzer I., Bennett E.M., Biggs R., Carpenter S.R., de Vries W., de Wit C.A. (2015). Planetary boundaries: Guiding human development on a changing planet. Science.

[4] Suding K., Higgs E., Palmer M., Callicott J.B., Anderson C.B., Baker M., Gutrich J.J., Hondula K.L., LaFevor M.C., Larson B.M.H. (2015). Committing to ecological restoration. Science.

[5] Theobald D.M. (2010). Estimating natural landscape changes from 1992 to 2030 in the conterminous US. Landsc. Ecol..

[6] Zhou H.C., Ma A.Z., Liu G.H., Zhou X.R., Yin J., Liang Y., Wang F., Zhuang G.Q. (2021). Reduced interactivity during microbial community degradation leads to the extinction of Tricholomas matsutake. Land Degrad. Dev..

[7] Cheng C., Gao M., Zhang Y., Long M., Wu Y., Li X. (2021). Effects of disturbance to moss biocrusts on soil nutrients, enzyme activities, and microbial communities in degraded karst landscapes in southwest China. Soil Biol. Biochem..

[8] Tucker C., Antoninka A., Day N., Poff B., Reed S. (2020). Biological soil crust salvage for dryland restoration: An opportunity for natural resource restoration. Restor. Ecol..

[9] Strickland M.S., Callaham M.A., Gardiner E.S., Stanturf J.A., Leff J.W., Fierer N., Bradford M.A. (2017). Response of soil microbial community composition and function to a bottomland forest restoration intensity gradient. Appl. Soil Ecol..

[10] Wang C., Zhang W., Zhao C., Shi R., Xue R., Li X. (2020). Revegetation by sowing reduces soil bacterial and fungal diversity. Ecol. Evol..

[11] Stanturf J.A., Palik B.J., Dumroese R.K. (2014). Contemporary forest restoration: A review emphasizing function. For. Ecol. Manag..

[12] Xu R., Zhao H., Liu G., You Y., Ma L., Liu N., Zhang Y. (2021). Effects of nitrogen and maize plant density on forage yield and nitrogen uptake in an alfalfa-silage maize relay intercropping system in the North China Plain. Field Crops Res..

[13] Zhou Z., Zhang Y., Zhang F. (2022). Abundant and rare bacteria possess different diversity and function in crop monoculture and rotation systems across regional farmland. Soil Biol. Biochem..

[14] Dong W.-H., Zhang S., Rao X., Liu C.-A. (2016). Newly-reclaimed alfalfa forage land improved soil properties comparison to farmland in wheat-maize cropping systems at the margins of oases. Ecol. Eng..

[15] Xu R., Zhao H., Liu G., Li Y., Li S., Zhang Y., Liu N., Ma L. (2022). Alfalfa and silage maize intercropping provides comparable productivity and profitability with lower environmental impacts than wheat-maize system in the North China plain. Agric. Syst..

[16] Hafner S., Kuzyakov Y. (2016). Carbon input and partitioning in subsoil by chicory and alfalfa. Plant Soil.

[17] Clement C., Sleiderink J., Svane S.F., Smith A.G., Diamantopoulos E., Desbroll D.B., Thorup-Kristensen K. (2022). Comparing the deep root growth and water uptake of intermediate wheatgrass (Kernza (R)) to alfalfa. Plant Soil.

[18] Jokela W., Posner J., Hedtcke J., Balser T., Read H. (2011). Midwest Cropping System Effects on Soil Properties and on a Soil Quality Index. Agron. J..

[19] Venter Z.S., Jacobs K., Hawkins H.J. (2016). The impact of crop rotation on soil microbial diversity: A meta-analysis. Pedobiologia.

[20] Xu H., Liu N., Zhang Y. (2022). Short-Term Snow Removal Alters Fungal but Not Bacterial Beta Diversity and Structure during the Spring Snowmelt Period in a Meadow Steppe of China. J. Fungi.

[21] Zhou J.Z., Deng Y., Shen L.N., Wen C.Q., Yan Q.Y., Ning D.L., Qin Y.J., Xue K., Wu L.Y., He Z.L. (2016). Temperature mediates continental-scale diversity of microbes in forest soils. Nat. Commun..

[22] Hall E.K., Bernhardt E.S., Bier R.L., Bradford M.A., Boot C.M., Cotner J.B., del Giorgio P.A., Evans S.E., Graham E.B., Jones S.E. (2018). Understanding how microbiomes influence the systems they inhabit. Nat. Microbiol..

[23] Jansson J.K., Hofmockel K.S. (2020). Soil microbiomes and climate change. Nat. Rev. Microbiol..

[24] Strickland M.S., Lauber C., Fierer N., Bradford M.A. (2009). Testing the functional significance of microbial community composition. Ecology.

[25] Waldrop M.P., Firestone M.K. (2006). Seasonal dynamics of microbial community composition and function in oak canopy and open grassland soils. Microb. Ecol..

[26] Li Y., Jia Z.J., Sun Q.Y., Zhan J., Yang Y., Wang D. (2016). Ecological restoration alters microbial communities in mine tailings profiles. Sci. Rep..

[27] Yan D.F., Mills J.G., Gellie N.J.C., Bissett A., Lowe A.J., Breed M.F. (2018). High-throughput eDNA monitoring of fungi to track functional recovery in ecological restoration. Biol. Conserv..

[28] Maul J., Drinkwater L. (2010). Short-term plant species impact on microbial community structure in soils with long-term agricultural history. Plant Soil.

[29] Zhao J., Xu Y., Peng L., Liu G., Wan X., Hua Y., Zhu D., Hamilton D.P. (2019). Diversity of anammox bacteria and abundance of functional genes for nitrogen cycling in the rhizosphere of submerged macrophytes in a freshwater lake in summer. J. Soils Sediments.

[30] Kamble P.N., Baath E. (2018). Carbon and Nitrogen Amendments Lead to Differential Growth of Bacterial and Fungal Communities in a High-pH Soil. Pedosphere.

[31] Rousk J., Baath E., Brookes P.C., Lauber C.L., Lozupone C., Caporaso J.G., Knight R., Fierer N. (2010). Soil bacterial and fungal communities across a pH gradient in an arable soil. ISME J..

[32] Yu Y., Zheng L., Zhou Y.J., Sang W.G., Zhao J.N., Liu L., Li C., Xiao C.W. (2021). Changes in soil microbial community structure and function following degradation in a temperate grassland. J. Plant Ecol..

[33] Wang C.Q., Xue L., Dong Y.H., Jiao R.Z. (2021). Effects of stand density on soil microbial community composition and enzyme activities in subtropical Cunninghamia lanceolate (Lamb.) Hook plantations. For. Ecol. Manag..

[34] Berendsen R.L., Pieterse C.M.J., Bakker P. (2012). The rhizosphere microbiome and plant health. Trends Plant Sci..

[35] Mendez M., Garcia D., Maestre F.T., Escudero A. (2008). More ecology is needed to restore Mediterranean ecosystems: A reply to Valladares and Gianoli. Restor. Ecol..

[36] Ge A.H., Liang Z.H., Xiao J.L., Zhang Y., Zeng Q., Xiong C., Han L.L., Wang J.T., Zhang L.M. (2021). Microbial assembly and association network in watermelon rhizosphere after soil fumigation for Fusarium wilt control. Agric. Ecosyst. Environ..

[37] Dong K., Yu Z., Kerfahi D., Lee S.S., Li N., Yang T., Adams J.M. (2022). Soil microbial co-occurrence networks become less connected with soil development in a high Arctic glacier foreland succession. Sci. Total Environ..

[38] Chen W., Wang J., Chen X., Meng Z., Xu R., Duoji D., Zhang J., He J., Wang Z., Chen J. (2022). Soil microbial network complexity predicts ecosystem function along elevation gradients on the Tibetan Plateau. Soil Biol. Biochem..

[39] Pržulj N., Malod-Dognin N. (2016). Network analytics in the age of big data. Science.

[40] Barberan A., Bates S.T., Casamayor E.O., Fierer N. (2012). Using network analysis to explore co-occurrence patterns in soil microbial communities. ISME J..

[41] Xue L., Ren H.D., Brodribb T.J., Wang J., Yao X.H., Li S. (2020). Long term effects of management practice intensification on soil microbial community structure and co-occurrence network in a non-timber plantation. For. Ecol. Manag..

[42] Yuan M.M., Guo X., Wu L.W., Zhang Y., Xiao N.J., Ning D.L., Shi Z., Zhou X.S., Wu L.Y., Yang Y.F. (2021). Climate warming enhances microbial network complexity and stability. Nat. Clim. Change.

[43] Bowman R.A. (1988). A Rapid Method to Determine Total Phosphorus in Soils. Soil Sci. Soc. Am. J..

[44] Xiao-Na L.I., Zhang W.W., Zhao C.Q., Song J.K., Shi R.S., Xue R.B., Wang C. (2019). Plant Diversity and Soil Physicochemical Properties in the Wasteland of Yanqing District. Acta Agrestia Sin..

[45] Caporaso J.G., Lauber C.L., Walters W.A., Berg-Lyons D., Huntley J., Fierer N., Owens S.M., Betley J., Fraser L., Bauer M. (2012). Ultra-high-throughput microbial community analysis on the Illumina HiSeq and MiSeq platforms. ISME J..

[46] McGuire K.L., Payne S.G., Palmer M.I., Gillikin C.M., Keefe D., Kim S.J., Gedallovich S.M., Discenza J., Rangamannar R., Koshner J.A. (2013). Digging the New York City Skyline: Soil Fungal Communities in Green Roofs and City Parks. PLoS ONE.

[47] Wagg C., Schlaeppi K., Banerjee S., Kuramae E.E., van der Heijden M.G.A. (2019). Fungal-bacterial diversity and microbiome complexity predict ecosystem functioning. Nat. Commun..

[48] Montoya J.M., Pimm S.L., Solé R.V. (2006). Ecological networks and their fragility. Nature.

[49] Liu M., Li X., Zhu R., Chen N., Ding L., Chen C. (2021). Vegetation richness, species identity and soil nutrients drive the shifts in soil bacterial communities during restoration process. Environ. Microbiol. Rep..

[50] Bienes R., Marques M.J., Sastre B., García-Díaz A., Ruiz-Colmenero M. (2016). Eleven years after shrub revegetation in semiarid eroded soils. Influence in soil properties. Geoderma.

[51] Zhang K., Li X., Cheng X., Zhang Z., Zhang Q. (2019). Changes in soil properties rather than functional gene abundance control carbon and nitrogen mineralization rates during long-term natural revegetation. Plant Soil.

[52] Huang C., Zeng Y., Wang L., Wang S. (2020). Responses of soil nutrients to vegetation restoration in China. Reg. Environ. Change.

[53] Li Q., Zhou D., Jin Y., Wang M., Song Y., Li G. (2014). Effects of fencing on vegetation and soil restoration in a degraded alkaline grassland in northeast China. J. Arid Land.

[54] Liang Y., Pan F., He X., Chen X., Su Y. (2016). Effect of vegetation types on soil arbuscular mycorrhizal fungi and nitrogen-fixing bacterial communities in a karst region. Environ. Sci. Pollut. Res..

[55] Hu P., Zhao Y., Xiao D., Xu Z., Zhang W., Xiao J., Wang K. (2021). Dynamics of soil nitrogen availability following vegetation restoration along a climatic gradient of a subtropical karst region in China. J. Soils Sediments.

[56] Zhu Y., Guo B., Liu C., Lin Y., Fu Q., Li N., Li H. (2021). Soil fertility, enzyme activity, and microbial community structure diversity among different soil textures under different land use types in coastal saline soil. J. Soils Sediments.

[57] Fu B., Qi Y., Chang Q. (2015). Impacts of revegetation management modes on soil properties and vegetation ecological restoration in degraded sandy grassland in farming-pastoral ecotone. Int. J. Agric. Biol. Eng..

[58] Qi Y., Yang F., Shukla M.K., Pu J., Chang Q., Chu W. (2015). Desert Soil Properties after Thirty Years of Vegetation Restoration in Northern Shaanxi Province of China. Arid Land Res. Manag..

[59] Lin J., Roswanjaya Y.P., Kohlen W., Stougaard J., Reid D. (2021). Nitrate restricts nodule organogenesis through inhibition of cytokinin biosynthesis in *Lotus japonicus*. Nat. Commun..

[60] Remigi P., Zhu J., Young J.P.W., Masson-Boivin C. (2016). Symbiosis within Symbiosis: Evolving Nitrogen-Fixing Legume Symbionts. Trends Microbiol..

[61] Xu H., Zhang Y., Shao X., Liu N. (2021). Soil nitrogen and climate drive the positive effect of biological soil crusts on soil organic carbon sequestration in drylands: A Meta-analysis. Sci. Total Environ..

[62] Wang K., Wang X., Fei H., Wan C., Han F. (2022). Changes in diversity, composition and assembly processes of soil microbial communities during Robinia pseudoacacia L. restoration on the Loess Plateau, China. J. Arid Land.

[63] Yang Y., Cheng H., Liu L., Dou Y., An S. (2020). Comparison of soil microbial community between planted woodland and natural grass vegetation on the Loess Plateau. For. Ecol. Manag..

[64] Zhang X.X., Wang L.J., Zhou W.X., Hu W., Hu J.W., Hu M. (2022). Changes in litter traits induced by vegetation restoration accelerate litter decomposition in Robinia pseudoacacia plantations. Land Degrad. Dev..

[65] Xu M.-p., Wang J.-y., Zhu Y.-f., Han X.-h., Ren C.-j., Yang G.-h. (2022). Plant Biomass and Soil Nutrients Mainly Explain the Variation of Soil Microbial Communities During Secondary Succession on the Loess Plateau. Microb. Ecol..

[66] Yang Y., Li T., Wang Y., Dou Y., Cheng H., Liu L., An S. (2021). Linkage between soil ectoenzyme stoichiometry ratios and microbial diversity following the conversion of cropland into grassland. Agric. Ecosyst. Environ..

[67] Zhang Y., Cao H., Zhao P., Wei X., Ding G., Gao G., Shi M. (2021). Vegetation Restoration Alters Fungal Community Composition and Functional Groups in a Desert Ecosystem. Front. Environ. Sci..

[68] Bastida F., Hernandez T., Albaladejo J., Garcia C. (2013). Phylogenetic and functional changes in the microbial community of long-term restored soils under semiarid climate. Soil Biol. Biochem..

[69] Lu Z.X., Wang P., Ou H.B., Wei S.X., Wu L.C., Jiang Y., Wang R.J., Liu X.S., Wang Z.H., Chen L.J. (2022). Effects of different vegetation restoration on soil nutrients, enzyme activities, and microbial communities in degraded karst landscapes in southwest China. For. Ecol. Manag..

[70] Araujo A.S.F., Borges C.D., Tsai S.M., Cesarz S., Eisenhauer N. (2014). Soil bacterial diversity in degraded and restored lands of Northeast Brazil. Antonie Van Leeuwenhoek.

[71] Li P., Zhang X., Hao M., Cui Y., Zhu S., Zhang Y. (2019). Effects of Vegetation Restoration on Soil Bacterial Communities, Enzyme Activities, and Nutrients of Reconstructed Soil in a Mining Area on the Loess Plateau, China. Sustainability.

[72] Xue L., Ren H.D., Li S., Leng X.H., Yao X.H. (2017). Soil Bacterial Community Structure and Co-occurrence Pattern during Vegetation Restoration in Karst Rocky Desertification Area. Front. Microbiol..

[73] Song S.Z., Xiong K.N., Chi Y.K., He C., Fang J.Z., He S.Y. (2022). Effect of Cultivated Pastures on Soil Bacterial Communities in the Karst Rocky Desertification Area. Front. Microbiol..

[74] Hu L., Li Q., Yan J.H., Liu C., Zhong J.X. (2022). Vegetation restoration facilitates belowground microbial network complexity and recalcitrant soil organic carbon storage in southwest China karst region. Sci. Total Environ..

[75] Liang C., Schimel J.P., Jastrow J.D. (2017). The importance of anabolism in microbial control over soil carbon storage. Nat. Microbiol..

