# Supplementary material for: Short-Term Vegetation Restoration Enhances the Complexity of Soil Fungal Network and Decreased the Complexity of Bacterial Network
Source: J Fungi (Basel). 2022 Oct 25;8(11):1122. doi: 10.3390/jof8111122 (PMC9695196; doi:10.3390/jof8111122)
Supplement: Supplementary file 1 [file jof-08-01122-s001.zip › jof-1953891-supplementary.pdf]

Effects of Short-Term Vegetation Restoration on Soil Microbial Diversity, Microbial Community Structure, and Microbial Network Complexity

Hengkang Xu[1], Chao Chen[1], Zhuo Pang[1], Guofang Zhang[1] Juying Wu[1] and Haiming Kan[1*]

[1]Institute of Grassland, Flowers and Landscape Ecology, Beijing Academy of Agriculture and Forestry Sciences (BAAFS), Beijing, China, No. 9 Shuguang Garden Middle Road, Haidian District

Correspondence: kanhaiming@hotmail.com

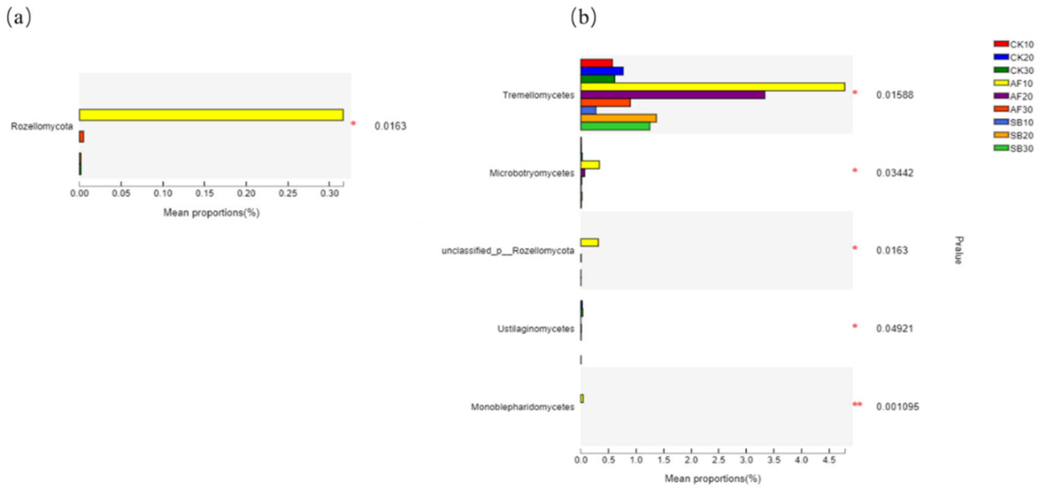

Figure. S1. Kruskal-Wallis test bar plot for fungal phyla **(a)** and classes **(b)** in in different restoration methods. Statistically significant differences are indicated with: * $P \leq 0.05$, ** $P \leq 0.01$.

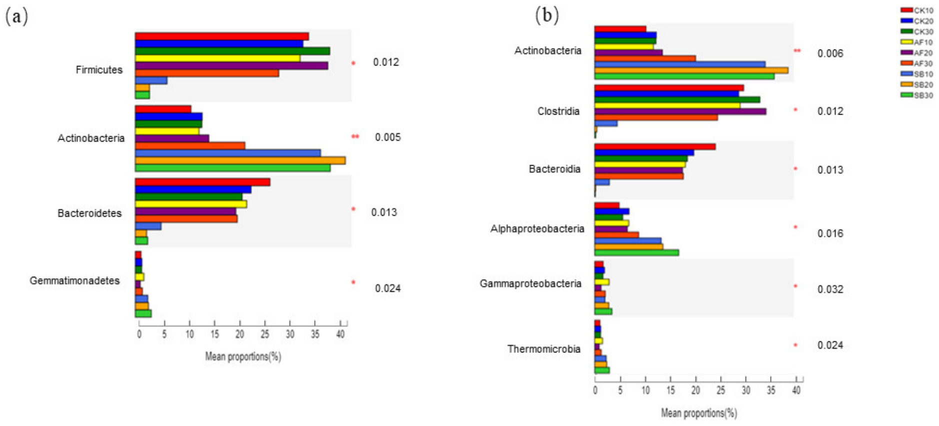

Figure. S2. Kruskal-Wallis test bar plot for bacterial phyla **(a)** and classes **(b)** in in

different restoration methods. Statistically significant differences are indicated with: * $P \leq 0.05$, ** $P \leq 0.01$.

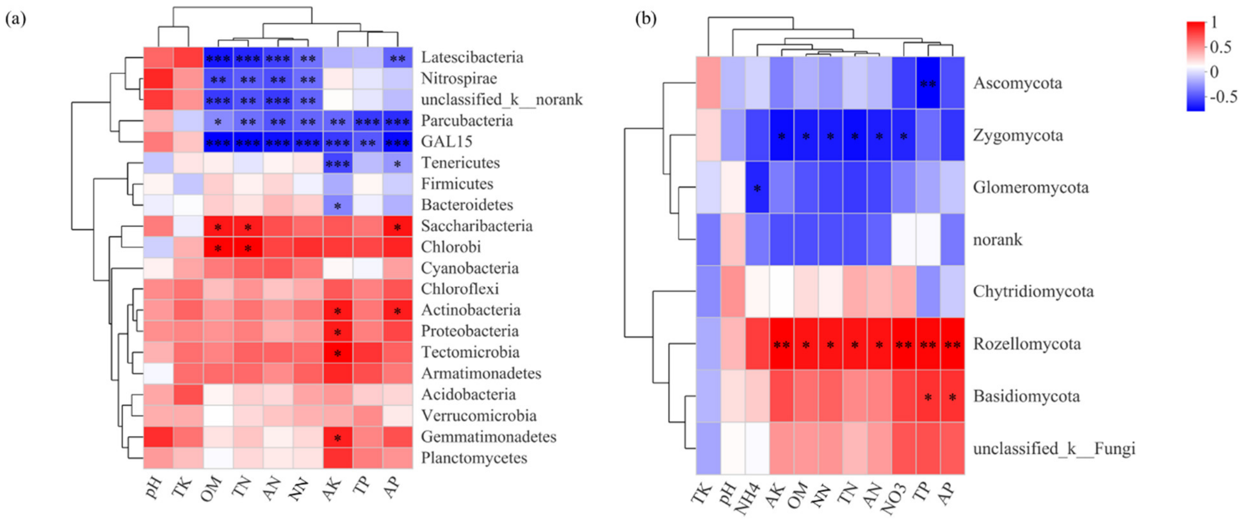

Figure. S3. Spearman correlation heatmaps of environment factors, biochemical properties and bacterial gene read numbers at the phylum (**a**) and fungal gene read numbers at the phylum (**b**) level. The color intensity in each panel indicates the relative correlation between soil properties and read numbers of each group. TK: Total potassium; OM, Organic matter; TN, Total nitrogen; AN: Ammonium nitrogen; NN: Nitrate nitrogen; AK: Available potassium; TP: Total phosphorus; AP: Available phosphorus; *$p \leq 0.05$, **$p \leq 0.01$, ***$p \leq 0.001$.
